# Effect of herbal toothpaste on the colour stability, surface roughness, and microhardness of aesthetic restorative materials—an in vitro study

**DOI:** 10.1038/s41405-024-00280-x

**Published:** 2024-12-17

**Authors:** Yashodhara Hazra, Arathi Rao, Srikant Natarajan, Suprabha Baranya Srikrishna

**Affiliations:** 1 Pediatric and Preventive Dentistry, Manipal College of Dental Sciences, Mangalore, Manipal Academy of Higher Education, Manipal, Udupi, Karnataka India; 2 Oral Pathology, Manipal College of Dental Sciences, Mangalore, Manipal Academy of Higher Education, Manipal, Udupi, Karnataka India

**Keywords:** Tooth brushing, Paediatric dentistry

## Abstract

**Objective:**

To evaluate the in vitro effects of two commonly used commercial herbal toothpastes (Dabur Meswak and Patanjali Dant Kanti) on the colour stability, surface texture, and microhardness of two commonly used aesthetic restorative materials, i.e., nanofilled composite and resin-modified glass ionomer cement (NFC and RMGIC).

**Materials and methods:**

The samples were brushed twice daily using two herbal toothpastes, Dabur Meswak (Dabur India Ltd) and Patanjali Dant Kanti (Patanjali Ayurved Ltd, India) and powered toothbrush. Atomic force microscopy, spectroscopy, and digital micro hardness testing were used to estimate the changes in the surface roughness, colour change, and hardness of the samples, respectively, at baseline and after six months.

**Results:**

Regarding colour change, a statistically significant difference (*P* < 0.05) was observed between baseline and six months in both the RMGIC and NFC for both herbal toothpastes. Both RMGIC and NFC had higher Ra values when brushed with either toothpaste, indicating a rough surface. The greatest increase in the mean difference in microhardness was observed for the Patanjali Dant Kanti toothpaste samples brushed on both NFC and RMGIC.

**Conclusion:**

In our work, herbal toothpaste increased surface roughness and microhardness and caused colour changes in the two most commonly used aesthetic restorative materials.

**Clinical relevance:**

Herbal toothpastes affect the surface texture of aesthetic dental restorative materials. Dental professionals may need to educate their patients to be cautious regarding the prolonged use of herbal toothpaste.

## Introduction

Oral hygiene maintenance is an important aspect of an individual’s overall well-being [1]. The desire to improve the quality of oral hygiene products and the shifting customer preferences for natural products have given rise to many types of herbal dentifrices [2, 3]. The size of the worldwide market for herbal toothpaste, which was 1.5 billion USD in 2018, is evidence of this [4]. Notably, in India, herbal toothpaste accounts for 21.0% (90.0%) of the majority (90.0%) of the total purchases in the Asia-Pacific region [5]. There is an increased influx of a variety of herbal toothpastes into the Indian market [6] and studies [7–9] have shown that toothpastes can affect the surface characteristics of teeth as well as restorative material.

Resin cements, such as resin-modified glass ionomer cement (RMGIC) and nanofilled composites (NFC), are the preferred restoration materials for the paediatric population due to their good aesthetics and longevity [10].

Colour stability, surface roughness and microhardness are key characteristics that determine the success of dental restorative materials in terms of its functionality, friction, wear resistance and aesthetic appeal and the cost-effectiveness of any material is directly associated with the longevity of the restoration [3, 11].

The increased use of herbal toothpastes makes it essential to assess whether these toothpastes, in addition to their therapeutic effects, have any detrimental effects on restorations.

Although studies have been conducted to determine the abrasive action of herbal toothpastes on enamel surfaces, there is a lack of evidence on the effect of herbal toothpastes on the surface characteristics of aesthetic restorations.

Hence, the present study was conceptualized to evaluate the effect of two commonly used commercial herbal toothpastes, Dabur Meswak (TP1) and Patanjali Dant Kanti (TP2), on the colour stability, surface texture, and microhardness of the aesthetic restorative materials RMGIC and NFC [4].

Dabur Meswak toothpaste is composed of pure meswak extracts, of the Miswak tree (Salvadore Persica’) containing Salvadorine and benzylisothiocyanate, which are responsible for its antibacterial activity [12]. Salvadora persica has been reported to contain a wide variety of organic and inorganic compounds in its extract. The organic compounds include glycosides, saponins, flavonoids, alkaloids, tannins, benzyl derivatives, phenolic compounds, and organic acids. Identified inorganic compounds include anionic substances such as fluoride, chloride, sulfate, thiocyanate, and nitrate [13].

Patanjali Dantkanti is composed of a combination of herbal extracts and essential oils (Table 1). *Anacyclus pyrethrum* (*A. pyrethrum*) is a wild species belonging to the family Asteraceae, which is used in traditional medicines. It has a wide spectrum of use such as analgesic, antimicrobial, antidepressant, immunostimulant, sialagogue, antioxidant, aphrodisiac to name a few [14].

**Table 1:** Herbal toothpastes used in the study and their contents [12, 23–26].

Product name	Ingredients	Details
Dabur Meswak	Pure extract of bark of Salvadore Persica (Meswak/Miswak), Calcium carbonate, Sorbitol, water, silica, sodium lauryl sulphate, flavour, cellulose gum, carrageenan, sodium silicate, PVM/MA copolymer, sodium saccharin, zinc gluconate, sodium benzoate, benzyl alcohol, P-thymol	The bioactive components of Salvadora persica are Silica, Tanins, Resins, Salvadorine, Essential oils, Sulphur, Vitamin C, Sodium bicarbonate, Calcium, Fluoride, Chloride N-benzyl-2phenylacetamide, Benzyl isothiocyanate
Patanjali Dantkanti	Anacyclus Pyrethrum, Azadirachta India, Xanthoxylum Alatum, Acacia Arabica, Mentha Spicata, Syzygium Aromaticum, Piper Sylvaticum, Barleria Prionitis, Mimusops Elengi, Embelia Ribes, Curcuma Longa, Salvadora Persica, Quercus Infectoria, Calcium Carbonate Base, Sodium Benzoate as reservative, Available Fluoride content: 924 PPM approximately	Anacyclus Pyrethrum is used for treatment of toothache. Azadirachta India has anti-inflammatory properties. Xanthoxylum Alatum has anti-inflammatory property and reduces the redness and swelling.

The research null hypotheses for this study was as follows: There is no difference in the colour stability, surface roughness, or microhardness of the RMGIC/NFC restorative material brushed with TP1 or TP2.

## Materials and methodology

This was an in vitro experimental study design with three arms for each restorative material. The sample size was calculated using G*Power software (version 17 March 2020—Release 3.1.9.7, Heinrich-Heine Dusseldorf University, Dusseldorf, Germany). With an alpha error of 5%, a power of 80%, and an effective difference of 1 [11], the sample size was determined to be 21 per group.

As per the standardized protocol [8, 15], specimens of RMGIC and NFC, 8 mm in diameter and 4 mm in height were prepared using a customized brass mould for both materials and measured using a digital caliper.

### RMGIC samples

RMGIC that was used in the study was GC Fuji II Light Cured Universal Restorative Gold Label and contained strontium glass, fluor-amino-silicate glass in the powder and aqueous solution of polycarboxylic acid, TEGDMA and HEMA in the liquid.

Following the manufacturer’s instructions, RMGIC powder and liquid at a ratio of 3.2:1 were mixed for 20–25 seconds to obtain a homogenous mixture. The material was then packed into the mould and condensed using a condenser. Curing of each sample was performed for 40 seconds. Mylar strips were placed on both sides of the mould to obtain smooth surfaces. The RMGIC samples were polished using a fine-grit diamond bur and 3 M Sof-Lex XT discs (3 M™ India Pvt Ltd.) for 10 seconds.

### NFC samples

NFC used was 3 M ESPE Filtek Z350XT (shade A2B) and is composed of BIS-GMA, UDMA, TEGDMA and bis-EMA resins. The fillers are a combination of non aggregated 20 nm silica filler, non aggregated 4–11 mn zirconia filler and aggregated zirconia/silica cluster filler (comprising of 20 nm silica and 4–11 nm zirconia particles).

The A2 shade of the composite resin was selected because it is a common shade used in clinical practice. An incremental technique (each increment was 2 mm thick) was used to fill the mould, and the mould was then cured for 40 seconds per increment. Mylar strips were placed on both sides of the mould to obtain a smooth surface. After this, all the samples were uniformly polished and finished using a series of polishing discs (Shofu polishing discs—coarse, medium, fine and super fine for 10 secs each) and polished for 10 sec using Enhance polishing kit (Dentsply India Private Limited) using an electric handpiece at 1500 rpm.

The samples were then stored in distilled water for 1 day at 37 °C. [15–17]. Curing of the RMGIC and NFC samples were done using a LED curing light (Bluephase® Ivoclar Vivadent, India). Intensity of curing unit used to cure the samples were verified with a calibrated radiometer (Demetron 100, Demetron Research Corp, USA) for every 5 specimens.

#### Blinding

All the specimens were numbered and randomly allocated into five groups to make the study scientifically robust. The samples were numbered and randomly assigned to their respective sub groups using a list of random numbers created by RANDOM.ORG [18]. The investigators were not blinded to the toothpaste as TP1 was white in colour and TP2 was light brown. The principal investigator (YH) who evaluated the colour change, surface roughness and microhardness and another investigator who evaluated the data (SN) were blinded to the samples and group to which they belonged.

Group 1 included the RMGIC samples, and group 2 included the NFC samples. Each of these groups was further subdivided into 3 arms of 7 samples each, with one arm brushed with TP 1, the second arm with TP 2 and the third arm as the control, brushed with distilled water.

Group 1 – RMGIC group (*n* = 21)

- Group 1a - RMGIC with TP1 (*n* = 7)
- Group 1b - RMGIC with TP2 (*n* = 7)
- Group 1c- RMGIC with distilled water (*n* = 7)

Group 2 – NFC group (*n* = 21)

- Group 2a- NFC with TP1 (*n* = 7)
- Group 2b – NFC with TP2 (*n* = 7)
- Group 2c - NFC with distilled water (*n* = 7)

#### Brushing method

Dabur Meswak (Dabur India Ltd) (TP1) and Patanjali Dant Kanti (Patanjali Ayurved Ltd, India) (TP2) were the two toothpastes used in the present study.

Oral B, Rechargeable electric tootbrush with round head with criss cross bristles powered by Braun was used. It oscillates at 8,800 movements per minute and 20,000 pulsations per minute. The toothbrush heads will rotate 45° to the right & back to 45° to the left, as well as oscillates back & forth. The head of the brush was stabilized and positioned over the samples such that the head of the toothbrush was in contact with the prepared specimen continuously and uniformly during the brushing. Separate brush were used for each subgroups and brushing was done for 40 seconds for each sample. Two cycles of tooth brushing were carried out per day with a gap of 12 hours. Specimens were individually subjected to 1500 cycles of brushing equating it to 216 mins of tooth brushing which is equivalent to 6 months of toothbrushing for 40 secs each, 2 times a day[19, 20].

Toothpaste slurries were prepared by mixing the toothpaste with distilled water at a ratio of 1:3 by weight. The samples were stored in distilled water between brushing cycles [11, 15, 16].

All the samples were evaluated for surface roughness, colour changes, and microhardness at baseline and after 6 months.

#### Assessment of outcomes

##### Color stability assessment

A spectrophotometer (X-rite i1PRO Spectrophotometer) was used to measure the colour change. The samples were air-dried and then further dried using blotting paper before testing [11, 21]. The top surface of the samples was marked with a pen on the edge and tested for colour change. The colour was assessed using a spectrophotometer to evaluate the lightness (L*), green‒red (a*), and blue‒yellow (b*) scores. The change in colour from baseline to the end of 6 months was also calculated from ΔE using the following formula:

DE* = [(DL*)2 + (Da*)2 + (Db*)2]1/2

The software Profile Maker 5.0.10 – Measure Tool was used to assess ΔE.

##### Surface roughness estimation

Atomic force microscopy (AFM) (Innova™, Bruker Corporation, Coventry, UK) was used to estimate the changes in the surface roughness in noncontact tapping mode with a probe length of 125 μm, a width of 40 μm, and a tip thickness of 3.4 μm.

**Fig. 1:**
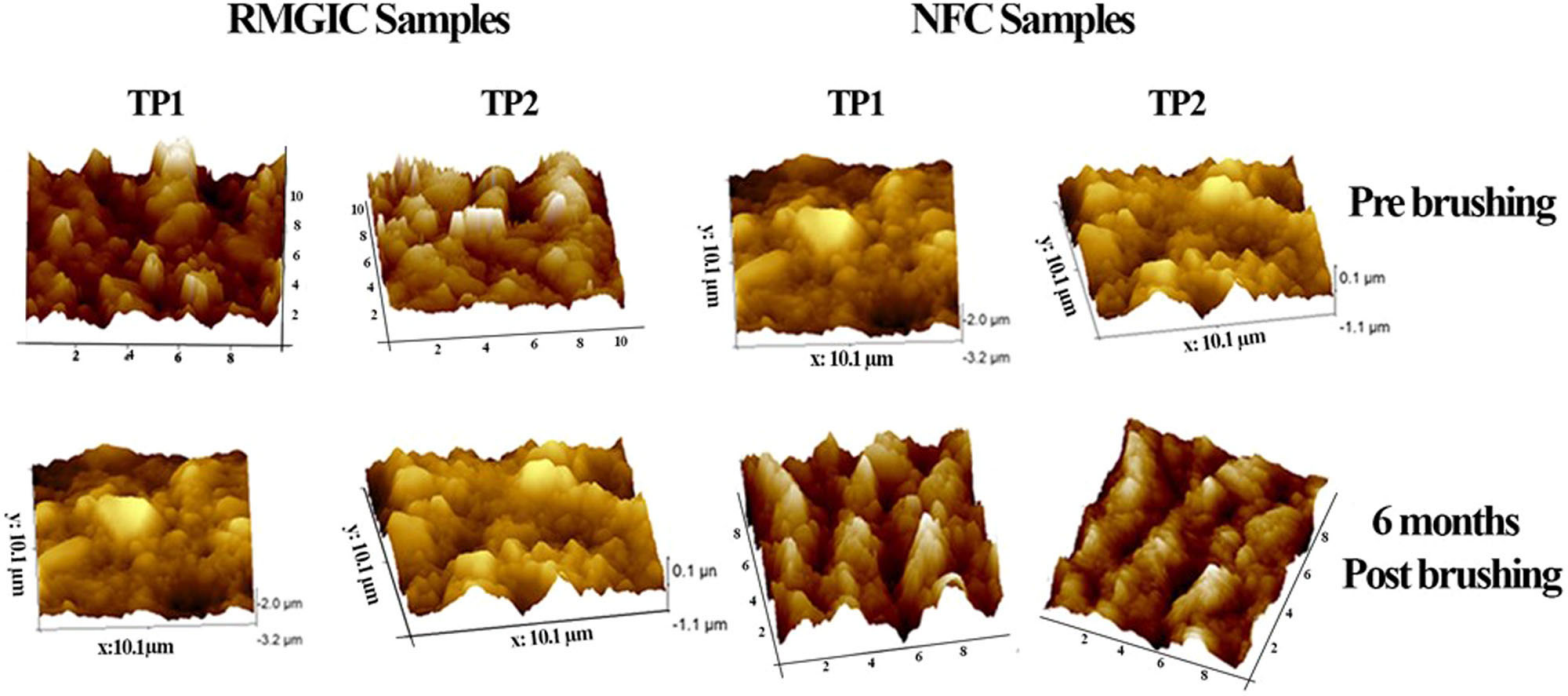
Atomic force microscopy images for estimation of surface roughness. Surface roughness was expressed as average Ra value (arithmetical average value of all absolute distances of the roughness profile) as well as peak-to-valley distance (Rp-v), expressed in nanometers.

The samples were air-dried before observation under AFM and placed on a sample mounting disk made of stainless steel 15 mm in diameter. The surfaces of the samples were scanned at three points (one point at the specimen’s centre, one point at the specimen’s perimeter, and one point at half distance between the specimen’s centre and perimeter), and the mean average value was obtained. Analysis of the samples was performed using Nanoscope Analysis Version 1.5 software (Bruker Innova). Three images were collected for each specimen on a compact disc. Images with 751 × 751 pixels were acquired with a scan size of 10 μm × 10 μm and a scan rate of 1.00 Hz (Fig. 1). The surface roughness was expressed as the average Ra value (arithmetical average value of all absolute distances of the roughness profile) [22].

##### Microhardness assessment

A digital microhardness tester (BHN Matsuzawa MMT-X, RHN Hitech India Equipment Pvt. Ltd., AI-TWIN) was used to test the microhardness of the samples. In the present study, the Vickers hardness of each specimen was measured at three different locations on the top surface of the sample by applying a load of 25 g for 20 seconds [1]. A diamond-shaped indentation was created at the end of each cycle of load application, which was used to read the values further. The average of the triplet measurements was taken.

#### Statistical analysis

The descriptive and inferential data were analysed using SPSS version 20.0 (Armonk, NY: IBM Corp). Based on normality, intragroup analysis was performed using an independent *t* test (baseline vs six weeks). Intergroup comparisons were performed using ANOVA followed by Tukey post hoc analysis. Descriptive statistics are presented as the mean ± standard deviation (SD). A *p* value less than 0.05 was considered to indicate statistical significance.

## Results

This in vitro study was conducted on RMGIC and NFC to determine the effect of herbal toothpastes on the color, surface roughness and microhardness of these restorative materials. The samples of each restorative material were allotted into 3 groups and brushed with two herbal toothpastes and distilled water. The composition of each toothpaste is given in Table 1 [12, 23–26].

The intra and intergroup comparison data for colour change, surface roughness and microhardness are given in Table 2.

**Table 2:** Intra and Inter-group comparison data for colour change, surface roughness and micro-hardness.

COLOUR CHANGE	Group	Baseline	6 months	Mean difference	F	*P* value
RMGIC + TP1	1a	31.93 ± 1.11	28.24 ± 1.55	3.69 ± 1.08	5.103	**0.000**
RMGIC + TP2	1b	31.31 ± 0.76	26.9 ± 1.97	4.41 ± 2.29	5.533	**0.000**
RMGIC + Distilled Water	1c	31.69 ± 1.06	31.43 ± 1.05	0.26 ± 0.17	0.457	0.656
NFC + TP1	2a	25.03 ± 1.2	27.61 ± 1.14	2.13 ± 1.77	−4.125	**0.001**
NFC + TP2	2b	24.57 ± 1.2	26.53 ± 1.36	2.44 ± 0.54	−2.853	**0.015**
NFC+Distilled Water	2c	31.39 ± 0.74	31.61 ± 0.9	−0.22 ± 0.25	−0.510	0.619
F		81.1	22.663	41.112		
*P* value		**<0.001**	**<0.001**	**<0.001**		
**SURFACE ROUGHNESS**						
RMGIC + TP1	1a	97.47 ± 9.59	114.33 ± 11.75	16.86 ± 9.88	−2.941	**0.020**
RMGIC + TP2	1b	89.79 ± 12.61	123.77 ± 18.35	33.99 ± 21.47	−4.038	**0.002**
RMGIC + Distilled Water	1c	100.48 ± 1.35	100.76 ± 1.27	0.28 ± 0.47	−0.406	0.692
NFC + TP1	2a	79.09 ± 8.17	120.29 ± 28.02	41.2 ± 24.71	−3.734	**0.003**
NFC + TP2	2b	87.9 ± 11.51	117.94 ± 19.36	30.04 ± 17.37	−3.529	**0.004**
NFC+Distilled Water	2c	79.27 ± 7.81	80.21 ± 8.16	0.94 ± 0.5	−0.221	0.829
F		17.742	12.402	13.863		
*p* value		**<0.001**	**<0.001**	**<0.001**		
**MICRO HARDNESS**						
RMGIC + TP1	1a	51.33 ± 1.43	52.87 ± 0.49	1.53 ± 1.35	−2.688	**0.020**
RMGIC + TP2	1b	51.68 ± 0.99	54.9 ± 1.35	3.22 ± 1.45	−5.103	**0.000**
RMGIC + Distilled Water	1c	51.56 ± 0.93	51.78 ± 0.67	0.22 ± 0.68	−0.502	0.625
NFC + TP1	2a	67.51 ± 1.85	75.4 ± 0.66	7.9 ± 2.23	−10.631	**0.000**
NFC + TP2	2b	68.62 ± 0.96	71.94 ± 1.04	3.32 ± 1.35	−6.185	**0.000**
NFC+Distilled Water	2c	65.78 ± 1.01	65.81 ± 1.03	0.03 ± 0.48	−0.063	0.951
F		**343.523**	**874.671**	**23.965**		
*p* value		**<0.001**	**<0.001**	**<0.001**		

Numbers in bold indicate statistically significant value.

### Colour stability

The lesser the colour change is, the greater the colour stability and the better the herbal toothpaste–restorative material combination for aesthetic reasons. An intragroup comparison was made for both the restorative material at baseline and at six months. The least colour change was observed in groups 1c and 2c (distilled water), 0.26 ± 0.17 (*p* = 0.656) and −0.22 + 0.25 (*p* = 0.619) respectively. The remaining groups exhibited notable colour changes that were statistically significant. The mean difference in colour change of 4.41 ± 2.29 was greatest in group 1b (RMGIC surface with TP2) (*p* = 0.000) followed by group 1a (RMGIC surface with TP1) with 3.69 ± 1.08 (*p* = 0.000). The mean colour differences in both NFC groups (Groups 2a and 2b) were 2.13 ± 1.77 (*p* = 0.001) and 2.44 ± 0.54 (*p* = 0.015) respectively.

### Surface roughness

Average Roughness, or Ra, is the most commonly specified surface texture parameter and unit of measurement was μm. It provides a general measure of the height of the texture across a surface and is the average of how far each point on the surface deviates in height from the mean height. Lower Ra is a desirable characteristic of any restorative material.

Both toothpastes caused significant changes in the surface roughness of both restorative materials. The least change in surface roughness of 16.86 ± 9.88 (*p* = 0.020) was observed in group 1a (RMGIC surface with TP1) and the greatest change of 41.2 ± 24.71 (*p* = 0.003) was observed in group 2a (NFC surface with TP1).

### Microhardness

The microhardness of the study samples progressively increased from baseline to six months in both the RMGIC and NFC groups treated with TP1 and TP2. The difference observed in the study samples brushed with distilled water was minimal and statistically non-significant. The greatest increase in the mean difference in hardness of 7.9 ± 2.23 (*p* = 0.000) was observed in group 2a (NFC surface with TP1) and the least hardness of 1.53 ± 1.35 (*p* = 0.020) was observed in group 1a (RMGIC surface with TP1).

### Intergroup comparison

Post hoc analysis revealed that there was no statistically significant difference between the two toothpastes with respect to colour change and surface roughness in the RMGIC or NFC. (*p* < 0.05) (Table 3). There was no significant difference in color change between TP1 and TP2 in RMGIC, with a mean difference of 1.34, a standard error of 0.73 (*p* = 0.462). Similarly, no difference in surface roughness was observed between the two toothpastes on RMGIC samples, with a mean difference of −9.44, a standard error of 9.00 (*p* = 0.898).

**Table 3:** Post hoc Tukey test for colour change, surface roughness and micro-hardness.

Parameter	Reference group	Comparison group	Mean difference	Standard error	*P* value	95%CI Upper bound	Lower bound
Colour Change	**1a**	**1b**	1.34286	0.73499	0.462	−0.8684	3.5541
		**2a**	0.62857	0.73499	0.955	−1.5827	2.8398
		**2b**	1.71429	0.73499	0.208	−0.4970	3.9256
		**2c**	−3.36714^*^	0.73499	**0.001**	−5.5784	−1.1559
	**1b**	**2a**	−0.71429	0.73499	0.924	−2.9256	1.4970
		**2b**	0.37143	0.73499	0.996	−1.8398	2.5827
		**2c**	−4.71000^*^	0.73499	**0.000**	−6.9213	−2.4987
	**1c**	**1a**	3.18571^*^	0.73499	**0.001**	0.9744	5.3970
		**1b**	4.52857^*^	0.73499	**0.000**	2.3173	6.7398
		**2a**	3.81429^*^	0.73499	**0.000**	1.6030	6.0256
		**2b**	4.90000^*^	0.73499	**0.000**	2.6887	7.1113
		**2c**	−0.18143	0.73499	1.000	−2.3927	2.0298
	**2a**	**2b**	1.08571	0.73499	0.680	−1.1256	3.2970
	**2c**	**2a**	3.99571^*^	0.73499	**0.000**	1.7844	6.2070
		**2b**	5.08143^*^	0.73499	**0.000**	2.8702	7.2927
Surface roughness	**1a**	**1b**	−9.44286	9.00542	0.898	−36.5363	17.6506
		**2a**	−5.95714	9.00542	0.985	−33.0506	21.1363
		**2b**	−3.61429	9.00542	0.999	−30.7078	23.4792
		**2c**	34.11429^*^	9.00542	**0.007**	7.0208	61.2078
	**1b**	**2a**	3.48571	9.00542	0.999	−23.6078	30.5792
		**2b**	5.82857	9.00542	0.986	−21.2649	32.9220
		**2c**	43.55714^*^	9.00542	**0.000**	16.4637	70.6506
	**1c**	**1a**	−13.56857	9.00542	0.662	−40.6620	13.5249
		**1b**	-23.01143	9.00542	0.135	−50.1049	4.0820
		**2a**	−19.52571	9.00542	0.277	−46.6192	7.5678
		**2b**	−17.18286	9.00542	0.414	−44.2763	9.9106
		**2c**	20.54571	9.00542	0.228	−6.5478	47.6392
	**2a**	**2b**	2.34286	9.00542	1.000	−24.7506	29.4363
	**2c**	**2a**	−40.07143^*^	9.00542	**0.001**	−67.1649	−12.9780
		**2b**	−37.72857^*^	9.00542	**0.002**	−64.8220	−10.6351
Microhardness	**1a**	**1b**	−2.03143^*^	0.49299	**0.003**	−3.5146	−0.5482
		**2a**	−22.53857^*^	0.49299	**0.000**	−24.0218	−21.0554
		**2b**	−19.07857^*^	0.49299	**0.000**	−20.5618	−17.5954
		**2c**	−12.94714^*^	0.49299	**0.000**	−14.4303	−11.4640
	**1b**	**2a**	−20.50714^*^	0.49299	**0.000**	−21.9903	−19.0240
		**2b**	−17.04714^*^	0.49299	**0.000**	−18.5303	−15.5640
		**2c**	−10.91571^*^	0.49299	**0.000**	−12.3989	−9.4325
	**1c**	**1a**	−1.09000	0.49299	0.258	−2.5732	0.3932
		**1b**	−3.12143^*^	0.49299	**0.000**	−4.6046	−1.6382
		**2a**	−23.62857^*^	0.49299	**0.000**	−25.1118	−22.1454
		**2b**	−20.16857^*^	0.49299	**0.000**	−21.6518	−18.6854
		**2c**	−14.03714^*^	0.49299	**0.000**	−15.5203	−12.5540
	**2a**	**2b**	3.46000^*^	0.49299	**0.000**	1.9768	4.9432
	**2c**	**2a**	−9.59143^*^	0.49299	**0.000**	−11.0746	−8.1082
		**2b**	−6.13143^*^	0.49299	**0.000**	−7.6146	−4.6482

Numbers in bold indicate statistically significant value.

*Denotes values that are statistically significant.

There was also no significant difference in color change between TP1 and TP2 on NFC samples, with a mean difference of 1.08, a standard error of 0.73 (*p* = 0.680). Similarly, no difference in surface roughness was observed between the two toothpastes on NFC samples, with a mean difference of 2.34, a standard error of 9.00 (*p* = 1.00).

However significant difference in microhardness was observed between TP1 and TP2 on RMGIC samples, with a mean difference of −2.03, a standard error of 0.49 (*p* = 0.003). Similarly, significant difference in microhardness was observed between the two toothpastes in NFC samples, with a mean difference of 3.46, a standard error of 0.49 (*p* = 0.000).

Intergroup comparisons between both TP1 and TP2 revealed significant differences in the microhardness of both restorative materials.

## Discussion

Multiple in vivo and in vitro studies have been conducted to establish the cleansing efficacy, antimicrobial properties, and therapeutic use of an array of herbal toothpastes on tooth surfaces [5, 27]. However, there is a paucity of literature assessing the interaction of herbal toothpaste with aesthetic restorative materias. Hence, in our study, a single investigator brushed the samples using a powered toothbrush to simulate real-life scenarios.

The roughness of a restorative material is dependent not only on the type of cleaning aid and its abrasive content but also on the technique used for finishing and polishing the restorative material [28]. We used a powered toothbrush to standardize the brushing technique and used “enhanced composite finishing kit” and “shofu” diamond polishing before polishing and finishing all the RMGIC/NFC samples, respectively.

### Colour changes

Instrumental techniques such as spectrophotometer have the advantage of eliminating subjective differences in the interpretation of colour and can differentiate the changes in colour below the threshold of the normal human eye. The CIEL*a*b system used in the present study can precisely sense and analyse even minute chromatic differences in colour [29, 30].

Furthermore, in our work, calibration of the unit was performed before testing. The samples were placed against the aperture on a white background to prevent potential absorption of light [11]. The ΔE value is expressed as the change in colour, i.e., ΔE < 1 indicates a change undetectable to the human eye, and a ΔE > 3 colour change is visible to all [31, 32].

The RMGIC demonstrated greater color change at the end of six months than did the NFC, which could be due to the increased wear resistance of the composite filler material rather than the RMGIC. Furthermore, TP2 demonstrated a greater degree of chroma change, which might be attributed to its contents.

### Surface roughness

Disruption of the surface texture results in plaque accumulation, which compromises not only the material transparency but also increases the risk for secondary caries [33, 34]. The surface roughness seen was greater with TP2 in the RMGIC group, and with TP1 in NFC group. Singla et al. [35] in their study on natural tooth enamel reported that Dabur Meswak toothpaste had significantly greater abrasiveness than Patanjali Dant Kanti and this was attributed to the presence of silica particles as abrasives in TP1 compared to calcium carbonate in TP2.

Akin to our reports on surface roughness, an in vitro study performed by Shah et al. [36] between Dabur Red toothpaste and Patanjali Dant Kanti on enamel blocks demonstrated that the greatest abrasive potential was observed in Patanjali Dant Kanti compared to Dabur Red. However, Aggarwal et al. [6], in their work on herbal and nonherbal toothpaste regarding the change in surface characteristics of enamel, concluded that Patanjali Dant Kanti toothpaste was less abrasive on the tooth surface than were Colgate and Dabur Red [7]. Athawale et al. [37] reported that Dabur caused the highest maximum abrasion on primary teeth compared to other commercial pastes used in the study. Hence, contradictory results prevail regarding the abrasive action of the most commonly used herbal toothpastes, i.e., Patanjali Dant Kanti and Dabur.

The overall change in surface roughness in either herbal toothpaste was greater in the NFC than in the RMGIC samples, which may be due to continuous wear by toothpaste caused by the filler particles of the composite material readily loosening, leading to increased surface roughness [38].

Microhardness is a vital property of restorative materials and is linked to their strength, proportionality limit, and wear resistance [39]. A comparison of the effects of TP1 and TP2 on the RMGIC and NFC, revealed that TP2 significantly increased the microhardness on RMGIC, while TP1 significantly increased the microhardness on NFC. It was also observed that TP1 and TP2 increased the microhardness of the NFC more than that of the RMGIC.

This study showed an increase in microhardness between baseline and the end of the sixth month in both toothpastes in their respective restorative material samples. Although Miswak, the main component of TP1, is found to contain fluoride [40, 41] and TP2 has been reported to contain 924 ppm fluoride along with calcium carbonate base [26], the role of fluoride in increasing the hardness of restorative materials could not be verified.

Other components in the toothpaste besides the abrasives, may also influence the abrasive potential of the toothpaste such as the pH, type of lubricants, type of detergents etc. [35]. In the present study, we were unable to analyze the specific components of the toothpastes, and relevant literature on this subject was limited.

The effect of toothpaste on surface characteristics varies depending on whether the study was conducted on enamel or restorative materials [42] and currently, no studies have evaluated the impact of herbal toothpastes on the surface characteristics of restorative materials. Therefore, the findings of the present study cannot be compared with existing literature.

Scope for future research would be to evaluate the effect of herbal toothpastes on other restorative materials such as nano-hybrid, micro-hybrid, bulk-fill, and flowable composites and to analyse the effects of each component of the herbal toothpastes.

### Limitations of the study

Details about the components that may cause discolouration or the presence of abrasives were not mentioned on the labels of the toothpastes and thus could not be identified in the present study. The study was an in vitro study carried out in a controlled environment for six months. Further long-term in vivo studies are required to determine their long term effects. The effects of herbal toothpaste on the surface properties of restorative materials was not compared with those of conventional toothpaste.

## Conclusion

With the above limitations, this study has the following conclusions:

1. The present evidence shows that herbal toothpastes affect the surface characteristics of dental aesthetic restorative materials and thus the null hypothesis was rejected.
2. Compared with TP1, TP2 caused greater colour change, surface roughness in RMGIC.
3. TP2 caused more colour change in the NFC. TP1 caused increased surface roughness and microhardness in NFC.
4. A comparison of the effects of TP1 and TP2 on the RMGIC, revealed that TP2 significantly increased the microhardness, while TP1 significantly increased the microhardness of the NFC.

## Clinical relevance

Herbal toothpastes have become easily available in supermarkets and their usage has increased among people. This study emphasizes the need to understand whether herbal toothpastes can cause changes in the surface characteristics of aesthetic restorative materials. Healthcare professionals need to be aware of these disadvantages and educate patients while providing aesthetic restorations.

## Supplementary information

CONSORT Checklist

## Data Availability

All data is available in the manuscript.
