# Supplementary material for: Effect of herbal toothpaste on the colour stability, surface roughness, and microhardness of aesthetic restorative materials—an in vitro study
Source: BDJ Open. 2024 Dec 17;10:95. doi: 10.1038/s41405-024-00280-x (PMC11652633; doi:10.1038/s41405-024-00280-x)
Supplement: Supplementary file 1 — CONSORT Checklist [file 41405_2024_280_MOESM1_ESM.pdf]

Modified CONSORT checklist of items for reporting in vitro studies of dental materials		
**Effect of herbal toothpaste on the colour stability, surface roughness, and microhardness of aesthetic restorative materials – an in vitro study**		
**Section/Topic**	**Checklist item**	**Reported on Page No.**
Abstract	Item 1. Structured summary of trial design, methods, results, and conclusions	2
**Introduction**		
Background and objectives	Item 2a. Scientific background and explanation of rationale<br>Item 2b. Specific objectives and/or hypotheses	3
**Methods**		
Intervention	Item 3. The intervention for each group, including how and when it was administered, with sufficient detail to enable replication	3-4
Outcomes	Item 4. Completely defined, pre-specified primary and secondary measures of outcome, including how and when they were assessed	6-7
Sample size	Item 5. How sample size was determined	4
Randomization Sequence generation	Item 6. Method used to generate the random allocation sequence	5-6
Allocation concealment mechanism	Item 7. Mechanism used to implement the random allocation sequence (for example, sequentially numbered containers), describing any steps taken to conceal the sequence until intervention was assigned	5
Implementation	Item 8. Who generated the random allocation sequence, who enrolled teeth,	5
Blinding	Item 9. If done, who was blinded after assignment to intervention (for example, care providers, those assessing outcomes), and how	5
Statistical methods	Item 10. Statistical methods used to compare groups for primary and	7
**Results**		
Outcomes and estimation	Item 11. For each primary and secondary outcome, results for each group,	7-8
**Discussion**		
Limitations	Item 12. Trial limitations, addressing sources of potential bias, imprecision,	11
**Other Information**		
Funding	Item 13. Sources of funding and other support (for example suppliers of drugs), role of funders	17
Protocol	Item 14. Where the full trial protocol can be accessed, if available	-

Reference: Clovis Mariano Faggion Jr.Guidelines for Reporting Pre-clinical In Vitro Studies on Dental Materials, J Evid Base Dent Pract 2012;12:182-189
